# Micromelt sampling of the glacier algal nutrient environment

**DOI:** 10.1093/femsec/fiaf098

**Published:** 2025-10-06

**Authors:** Madeleine Lewis, Emily L M Broadwell, Jasmin L Millar, Elizabeth R Thomas, Patricia Sanchez-Baracaldo, Christopher J Williamson

**Affiliations:** Bristol Glaciology Centre, School of Geographical Sciences, University of Bristol, Queens Avenue, Bristol, BS8 1QU, United Kingdom; British Antarctic Survey, High Cross, Madingley Road, Cambridge, CB3 0ET, United Kingdom; Department of Environmental Science, Aarhus University, Nordre Ringgade 1, 8000 Roskilde, Denmark; School of Earth and Environmental Sciences, Cardiff University, Park Place, Cardiff, CF10 3AT, United Kingdom; British Antarctic Survey, High Cross, Madingley Road, Cambridge, CB3 0ET, United Kingdom; Bristol Glaciology Centre, School of Geographical Sciences, University of Bristol, Queens Avenue, Bristol, BS8 1QU, United Kingdom; Bristol Glaciology Centre, School of Geographical Sciences, University of Bristol, Queens Avenue, Bristol, BS8 1QU, United Kingdom

**Keywords:** algal bloom, Arctic, glacier algae, ice, oligotrophic, supraglacial

## Abstract

Zygnematophycean “glacier algae” form extensive blooms on ablating glacier surfaces despite the ultra-oligotrophic conditions apparent. Previous work has postulated that this oligotrophic bloom paradox is due to (i) lower nutrient requirements of glacier algae, (ii) efficient uptake and storage of the nutrients available, and/or (iii) ineffective characterisation of the actual nutrient environment that glacier algae experience. We investigate the latter here by directly sampling the thin (∼2 mm) melt water film in which glacier algal cells reside across three glaciers in Svalbard during the 2023 melt season, comparing to outcomes from more typical bulk ice sampling techniques. Micromelt samples generally contained increased concentrations of ammonium (NH_4_^+^), nitrate (NO_3_^−^), nitrite (NO_2_^−^), and phosphate (PO_4_^3−^), though trends were not uniform, and concentrations remained well within oligotrophic levels. Several major ion species were significantly increased in micromelt fractions as compared to bulk samples, indicating aeolian deposition and marine aerosol influences on the glacier algal environment. In turn, enhanced micromelt dissolved organic carbon concentrations (DOC) indicated likely DOC delivery by glacier algae to the microbial food web from the onset of bloom formation. Taken together, datasets reveal new fine-scale heterogeneity in the glacier algal meltwater environment.

## Teaser

Glacier algae survive and form blooms in the thin film of melt water covering the surface ice of Arctic glaciers despite ultra-low nutrients.

## Introduction

Zygnematophycean “glacier algae” are ubiquitous across the cryosphere, forming widespread blooms in melting surface ice during summer melt seasons, analogous to phytoplankton blooms in marine environments (Takeuchi 2001, Di Mauro et al. 2020, Williamson et al. 2021, Liu et al. 2022, Millar et al. 2024). Blooms initiate following snow-line retreat (Tedstone et al. 2017) with the coincidence of liquid water, sunlight, and nutrient resources driving the growth and proliferation of glacier algal cells in the upper melt water film that surrounds melting surface ice crystals (Holland et al. 2019). On the Greenland Ice Sheet, population doubling times have been estimated at 3.75–5.5 days (Stibal et al. 2017, Williamson et al. 2018), with maximal cell densities recorded at ∼10^5^ cells mL^−1^ of meltwater during major bloom years (Yallop et al. 2012, Stibal et al. 2017, Williamson et al. 2018, 2020). Algal blooms are apparent on valley glaciers across the cryosphere, with cell densities reported as 8.95 × 10^5^ cells mL^−1^ of meltwater on Morteratsch glacier, Switzerland, during the record 2022 melt year (Millar et al. 2024). Given that an abundance of heavily pigmented glacier algal cells within surface ice directly exacerbates energy absorbance and melt (e.g. Stibal et al. 2017, Williamson et al. 2019, Di Mauro et al. 2020), understanding controls on bloom formation and proliferation remains a priority (Holland et al. 2019, Millar et al. 2024).

The abundance of glacier algae achieved within surface ice during blooms presents something of a paradox, recently termed “the oligotrophic bloom paradox” (Millar et al. 2024), because of the ultra-low-nutrient composition (generally defined as < 1 µmol L^−1^ NO_3_^−^ or NO_2_^−^, and <0.1 µmol L^−1^ PO_4_^3−^) of the ice matrix in which glacier algae proliferate (Holland et al. 2019, Williamson et al. 2019, Millar et al. 2024). For example, Holland et al. (2019) and Williamson et al. (2021) reported well-developed glacial agal blooms with considerable biomass on the Greenland Ice Sheet despite ambient nutrient concentrations of ammonium, nitrate, and phosphate often below or around detection limits (<1 µM) (Williamson et al. 2018, 2021). Similarly, Millar et al. (2024) reported highly abundant glacier algal communities thriving within oligotrophic ice on Morteratsch glacier, Switzerland, where nutrient concentrations ranged 0.22–4.36 µmol L^−1^ NH_4_^+^, 0.04–1.38 µmol L^−1^ NO_3_^−^, and 0.22–0.85 µmol L^−1^ PO_4_^3−^. They further tested for potential bottom-up control of glacier algal assemblages sampled from their site through inorganic nutrient spiking incubations and were unable to identify any inorganic macro nutrient limitation even after 5 days of incubation (Millar et al. 2024), reflecting findings from similar studies conducted in Greenland (McCutcheon et al. 2021, Halbach et al. 2025). Findings to-date thus suggest that certainly at the point of sampling, glacier algae do not appear to be macronutrient limited within surface ice environments of both mountain glacier and larger ice sheet systems (Millar et al. 2024, Halbach et al. 2025).

**Figure 1.**
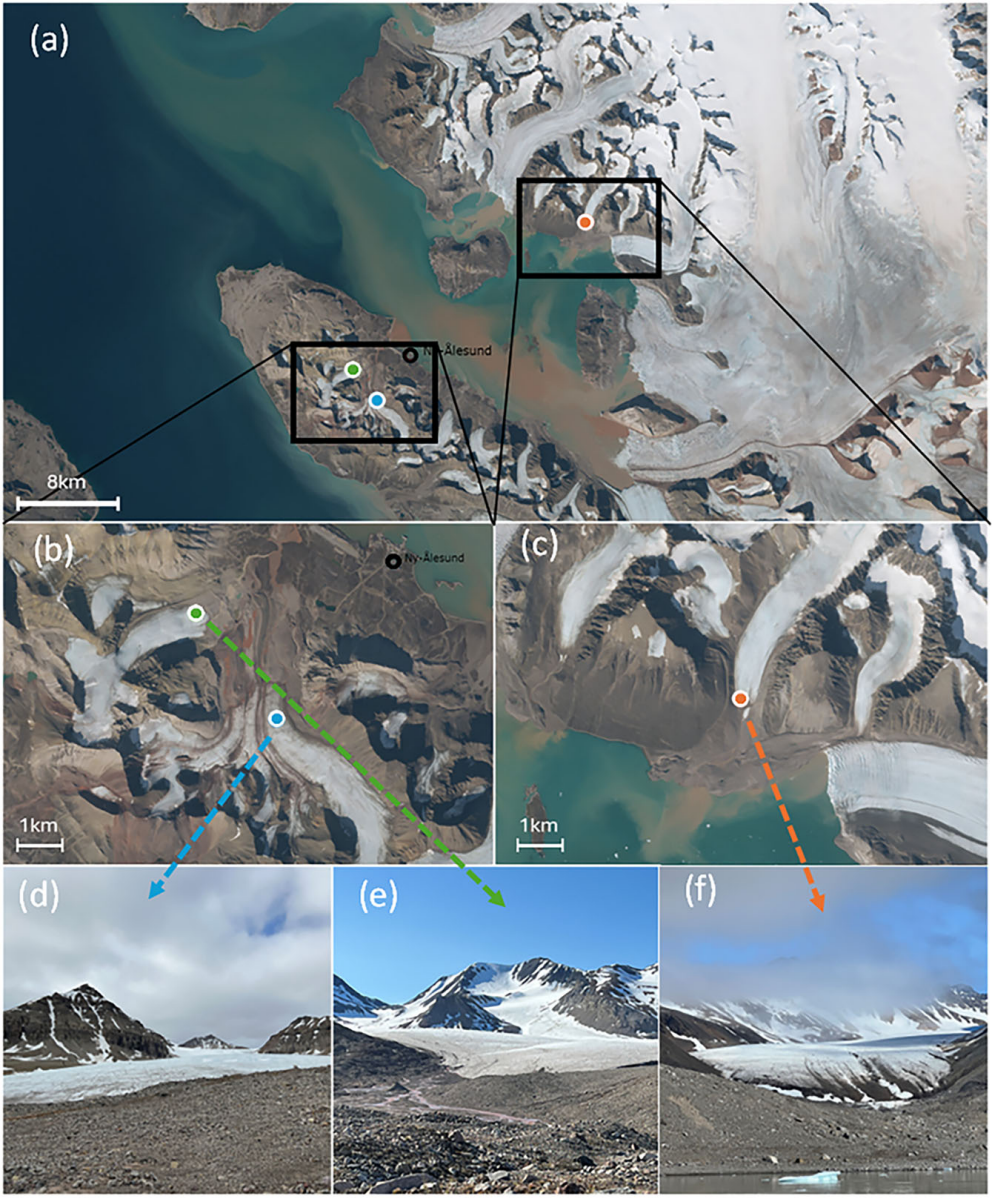
Glacier sampling sites in Svalbard, satellite photography of Kongsfjord and Ny Ålesund during summer ablation period 2023. (a) Vestre Brøggerbreen (78.914196°N, 11.754798°W) sampled 10 and 21 July 2023. (b) Austre Brøggerbreen (78.897387°N, 11.833194°W) sampled 19 and 25 July 2023. (c) Feiringbreen (79.006498°N, 12.447865°W) sampled 14 and 22 July 2023 (TopoSvalbard—Norsk Polarinstitutt, 2023.).

**Figure 2.**
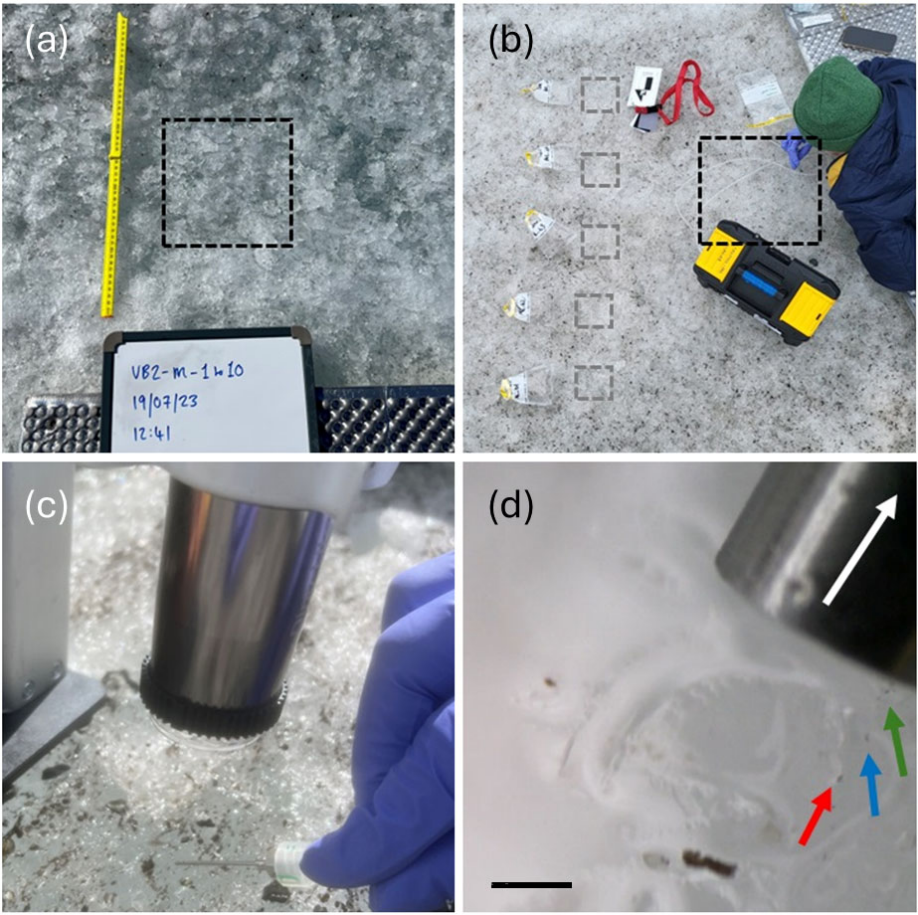
Supraglacial sampling method comparison (a) supraglacial sample site demarked by a black square (20 cm h^−1^ × 20 cm w^−1^ × ∼2 cm d^−1^). (b) Set-up of bulk samples (*n* = 5) and micromelt samples taken using a peristaltic pump and wide bore needle, with the sampled area indicated by gray and black squares, respectively. (c) Traditional bulk sampling collects both ice and water present in the ablating glacier surface via scoop. (d) *In situ* microscope image of algal cells (indicated by colored arrows) being suctioned up into a needle (white arrow) for liquid-only micromelt sampling, scale bar is 200μm.

Explanations offered for the disparity between glacier algal proliferation and almost unmeasurable nutrient concentrations within glacier surface ice include adaptation of glacier algae to their low nutrient environment through overall lower cellular macronutrient requirements (Williamson et al. 2021, Halbach et al. 2025), efficient uptake and storage of those scarce nutrients available within the system (Barcytė et al. 2020, Halbach et al. 2025), and/or poor characterisation of the actual nutrients available to glacier algae within melting surface ice (Millar et al. 2024). The latter stems from non-trivial complexities of sampling supraglacial surface ice and its associated microbial communities. Glacier algae reside within the thin melt water film (micromelt) that coats melting ice crystals of the weathered ablating ice of glaciers (Williamson et al. 2019, see Figs 1 and 2). This ice surface, termed the weathering crust, is highly heterogenous and dynamic relative to the surface energy balance and glacier algal loading (Müller and Keeler 1969, Cook et al. 2019, Tedstone et al. 2020). To-date, the traditional technique for sampling this ice has been to manually remove (using ice axe, ice saw or similar) a small area, melt this slowly, and take subsamples for algal counts and nutrient measurements. A potential problem with this “bulk” sampling method is dilution of the actual thin melt water film by the dominant solid-ice fraction as the sample is melted, which could cause underestimation of both true glacier algal abundance per unit melt water and aqueous nutrient concentrations. For example, we might expect enhanced nutrient concentrations within the thin melt water film inhabited by glacier algae due to processes of ice solute exclusion, whereby solutes are excluded from ice matrices during the freezing process and/or released early in the melt process, serving to concentrate solutes in the available melt water (Clarke et al. 2013). This more concentrated melt fraction may not be detectable by typical “bulk” sampling approaches. Glaciers are also open systems that receive exogenous inputs reflective of the continental crust in the form of mineral dust, e.g. Saharan dust events, and nearby water masses via marine aerosol e.g. from nearby fjords such as Kongsfjord (Dumont et al. 2023); presently, it is unknown how these influence glacier algal blooms.

In this study, we deploy micro-sampling techniques to directly characterise the thin melt water film inhabited by glacier algae across three high-Arctic glaciers in Svalbard, at the start of the 2023 melt season, and couple this with traditional “bulk” sampling approaches to contrast the biogeochemical outcomes of these two techniques. We show here that while the glacier algal micro-environment remains oligotrophic, micromelt sampling highlights a greater heterogeneity in macronutrient, major ion, and dissolved organic carbon (DOC) concentrations as compared to results from bulk-sampling techniques, and serves to unravel more of the two-way interactions apparent between glacier algae and their aqueous environment.

## Materials and methods

### Field site and sampling locations

We examined glacier algal blooms on three glaciers that represent a variety of typical high Arctic westerly glaciers in Svalbard Archipelago, near Ny Ålesund, during July 2023: Vestre Brøggerbreen (VB, 78.91°N, 11.75°E), Austre Brøggerbreen (AB, 78.89°N, 11.83°E), and Feiringbreen (FB, 79°N, 12.44°E) (Fig. 1, Supplementary Table 1). Vestre Brøggerbreen and Austre Brøggerbreen are both valley glaciers situated in the Bayelva basin to the south and south-west of Ny Ålesund research station, that feed into Kongsfjord and have north-east and north-west aspects respectively. Feiringbreen is also a valley-type glacier situated across the fjord, with a southerly aspect and steeper slope. All glaciers were visited twice over 2 weeks (VB 6 July 2023 and 19 July 2023; AB 14 July 2023 and 25 July 2023; FB 10 July 2023 and 22 July 2023) to characterise the progression of early ablation season dynamics. During each visit, supraglacial surface ice was sampled using both novel micro-sampling as well as traditional bulk sampling techniques (Fig. 1, Supplementary Table 1) for the determination of glacier algal abundance and aqueous geochemistry.

#### Micromelt sampling

To sample the melt water fraction of the upper supraglacial weathering crust (hereafter termed micromelt samples), a sterile 21-gauge needle was attached to a variable speed peristaltic pump (DIPump550, Kamoer, Shanghai, China) and used to directly sample the thin surface water film coating melting ice crystals (Lewis 2024, see Fig. 2). Sample collection number varied with field conditions and access, ranging from 5 to 14 (see Supplementary Table 1 for further information). *In situ* imaging of the ice surface using a digital microscope (Dino-Lite, AM7915MZT and RK-10A stand, Absolute Data Services Ltd) was done to confirm the presence of algal cells at the sample sites. Prior to sample collection, a 10-gauge wide-bore needle was used to rinse the micro-sampling system and pre-acid-washed high-density polyethylene (HDPE) sample collection bottles with water from a supraglacial stream within the sample vicinity. Subsequently, sterile 21-gauge needles were used to collect micromelt from the ice surface, taking care not to sample the main water table that lay deeper into the weathering crust. This sampling design was employed to characterise the aqueous geochemistry of the upper surface ice micro-habitat above the main water table that glacier algae are consistently observed to inhabit, though it is likely some glacier algal cells are washed into the underlying water table and transported through the weathering crust under some conditions (Irvine‐Fynn 2022). Given the practicalities of this fine-scale micro-melt sampling method, sample volumes collected ranged ∼1–15 ml across all samples depending on the abundance of melt water coating ice crystals at the day/time of sampling. All samples were taken between 12:00 and 18:00 on each sampling day.

#### Bulk sampling

To provide a comparison to micromelt sampling, traditional “bulk” sampling approaches (hereafter termed bulk samples) were conducted in parallel, mimicking the sample-melt-measure approach employed by studies to-date (*n*_bulk_ = 7–22, see Supplementary Table 1). For this, areas of the supraglacial ice surface were sampled directly adjacent to all micromelt samples on each glacier. For each sample, a 20 cm × 20 cm × 2 cm depth patch of ice was collected directly into a sterile Whirl-Pak bag using standard sampling techniques (Williamson et al. 2018). Before collection, metadata on slope, aspect, elevation, GPS position, and an RGB image were taken using the Phyphox app on an Apple iPhone 12 adjacent to sampling areas to avoid contamination. Before collection, metadata on slope, aspect, elevation, GPS position and an RGB image were taken using the Phyphox app on an Apple iPhone 12 (Staacks et al. 2018) adjacent to sampling areas to avoid contamination. All bulk samples were transported back to the NERC Arctic Research Station.

#### Sample processing

Micromelt samples were processed within 24 h of collection at the NERC Arctic Station, with bulk samples melted overnight in the dark over 1–2 days at 4°C and homogenised prior to further processing. For both micromelt and bulk samples, ∼1 ml of each sample was aliquoted into individual 1.5-ml Eppendorf tubes and fixed using Lugol’s solution for subsequent glacier algal cell counts at The University of Bristol, UK. A known volume of the sample was then filtered through a pre-combusted (450 °C for 5 h) 47-mm diameter GF/F filter (0.7-µm retention, Whatman, Maidstone, UK) with the filtrate stored in a pre-acid-washed HDPE bottle at −20°C for subsequent inorganic (ammonium [NH_4_^+^], nitrate [NO_3_^−^], nitrite [NO_2_^−^], and phosphate [PO_4_^3−^]) and organic (total organic carbon, TOC, and total nitrogen, TN) aqueous geochemistry quantification at The University of Bristol, UK; see Holland et al. (2019).

The cellular abundance (cells mL^−1^) of glacier algae (*Ancyclonema nordenskioldii* and *Ancyclonema alaskana*) and unidentified snow algal species were quantified for all micromelt and bulk samples by counting cells on a modified Fuchs Rosenthal Haemocytometer (0.2 mm × 1/16 mm^2^; Hawksley, Lancing, UK) using a bright field Olympus BX41 microscope (Germany) (Hillebrand et al. 1999). Images of each sample were taken at 10× and 40× magnification with a MicroPublisher 6 CCD camera attachment (Teledyne Photometrics, USA), from which cells were counted.

### Aqueous geochemistry

Aqueous concentrations of NH_4_^+^, NO_2_^−^, NO_3_^−^, and PO_4_^3−^ were derived for all micromelt and bulk ice samples spectrophotometrically using a Gallery Plus Discrete Photometric Analyser (Thermo Fisher Scientific, UK). The limit of detection (LoD) for all nutrients was determined by the mean concentration plus three times the standard deviation of calibration blanks (*n* = 3). LoDs were 0.09 (NH_4_^+^), 0.02 (NO_2_^−^), 0.20(NO_3_^−^), and 0.14 μmol L^−1^ (PO_4_^3−^). Precisions were ±2.7% (NH_4_^+^), ±0.3% (NO_2_^−^), ±0.5% (NO_3_^−^), and ±0.8% (PO_4_^3−^), as determined by comparison with diluted 71.43 mmol L^−1^ NH_4_^+^-N, NO_2_^−^-N, and NO_3_^−^-N and diluted 32.29 mmol L^−1^ PO_4_^3−^-P certified stock standards to a concentration of 3.6 (NH_4_^+^ and NO_2_^−^), 2.9 (NO_3_^−^), and 6.1 μmol L^−1^ (PO_4_^3−^) (Sigma TraceCERT^®^).

Aqueous concentrations of Cl^−^, SO_4_^2−^, Na^+^, K^+^, Mg^2+^, Ca^2+^ were analysed via a Dionex ICS-5000 Ion Chromatography (Thermo Fisher Scientific, UK). Standards containing all investigated ions were analysed and all replicate samples had a standard deviation <10%. To allow iconic activity comparison major ion datasets are reported in micro equivalents per litre (µeq L^−1^) (Fortner et al. 2005, Bagshaw et al. 2013). To evaluate the relative importance of marine aerosols or particulates as the source of solutes to micromelt and bulk samples, mean enrichment factors were calculated for each ion after Bagshaw et al. (2013). For this, the concentration of each ion species (X) relative to the concentration of Cl^−^ in its sample (ice), was ratioed with its concentration in seawater relative to seawater Cl^−^ (EF_X_ = (X_ice_ /Cl^−^_ice_)/(X_seawater_ /Cl^−^_seawater_)). Enrichment factors of 1 thus indicated no enrichment, > 1.2 demonstrated enrichment, and < 0.8 demonstrated depletion relative to sea salt.

Micromelt and bulk sample filtrate was also analysed for TOC and TN concentrations via a TOC/TN Organic Carbon Analyser (Shimadzu, UK). Non-purgeable organic carbon was measured after the acidification of samples with 9 N sulfuric acid and catalytic combustion at 720 °C as CO_2_. TN was measured as NO by chemiluminescence. The LoD was 13.43 (TOC) and 34.5 μmol L^−1^ (TN), and precision was ±1.3% as determined by comparison with diluted 41.7 mmol L^−1^ TOC-certified stock standards to a concentration of 2.5 mmol L^−1^ and ±1.7% by comparison with diluted 14.3 mmol L^−1^ TN certified stock standards to a concentration of 3.6 mmol L^−1^ (Sigma TraceCERT^®^).

### Data analysis

R. v.4.9.4 (Team 2022) was used to analyse and plot all data. All datasets were tested for normality (Shapiro–Wilk test, Bartlett’s test, skew, kurtosis) and compared statistically using the appropriate tests. Parametric data were analysed via paired *t*-test and analysis of variance, with *post hoc* Tukey Honest Significant Difference applied where appropriate. Non-parametric data were analysed via Mann–Whitney U/Wilcoxon rank sum test (MWU) or Kruskal–Wallis rank sum test.

## Results and discussion

**Figure 3.**
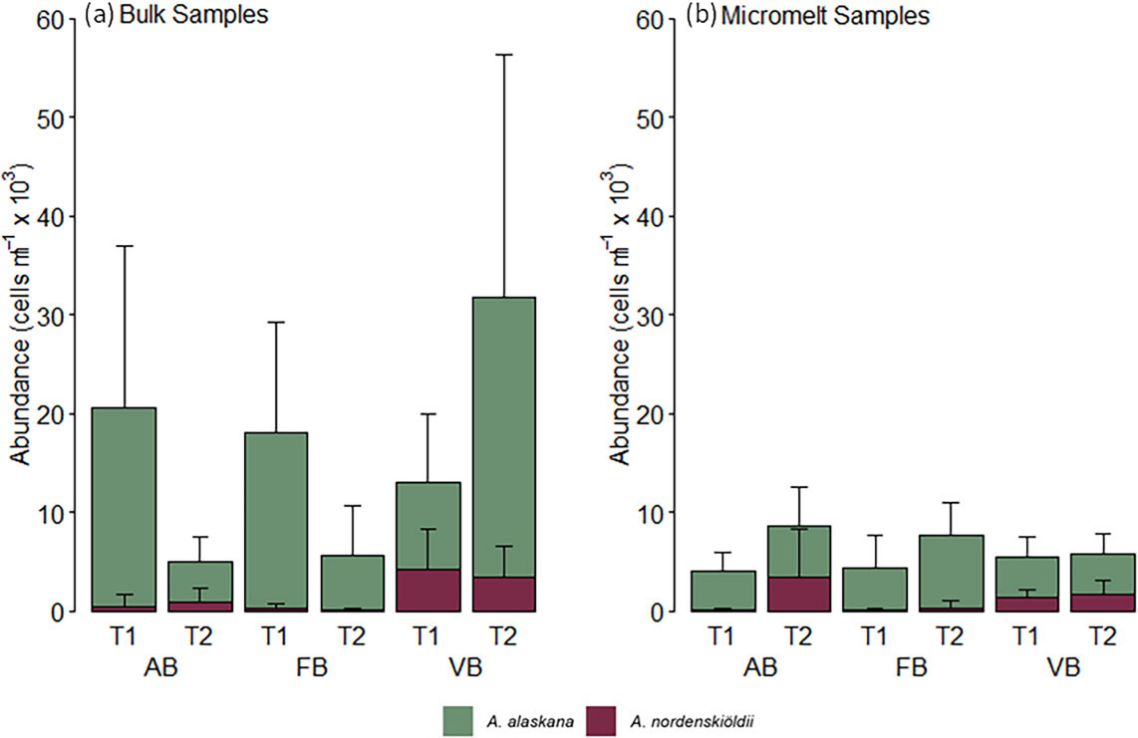
*Ancylonema* spp. abundance across three glaciers: Austre Brøggerbreen (AB), Feiringbreen (FB), and Vestre Brøggerbreen (VB) over two timepoints (T1, T2) approximately 2 weeks apart. Comparison of (a) bulk sampling method (*n* = 10) and (b) micromelt sampling method (*n* = 6**–**10).

Glacier algae were present across all sites and sampling times during this study, with abundance ranging 3.12 × 10^3^ to 5.34 × 10^5^ cells ml^−1^ across all data (Fig. 3). *Ancylonema* spp. dominated the supraglacial ice, with *A. alaskana* comprising the majority of the algal population across all glaciers (82%) followed by *A. nordenskioldii* (17.9%). These data are consistent with observations of glacier algal communities across myriad glacier and ice sheet systems, with as yet unknown controls on the relative frequency of glacier algal species apparent (Yallop et al. 2012, Williamson et al. 2018, 2021, Millar et al. 2024). While glacier algae were observed at all sampling locations and times, it was clear from field observations that the early-melt-season sampling conducted here corresponded to early phases of potential glacier algal blooms across the three glaciers. This was evidenced by the presence of remaining snowpack areas within glacier ablation zones and lack of conspicuous glacier algal bloom observable by eye. Unidentified algae and snow algal cysts were also present across all samples at concentrations ranging 0–(1.25 × 10^4^) cells ml^−1^, though we exclude these from subsequent discussion given the focus of this study.

Micromelt versus bulk sampling techniques resulted in significant differences in final glacier algal cell counts during this study (MWU, *P <* 0.05), with the latter bulk method consistently recovering greater glacier algal abundance across glaciers and sampling times (Fig. 3). For example, glacier algal abundance on the first date of sampling at Austre Brøggerbreen averaged 20.8 ± 16.8 × 10^3^ cells ml^−1^, as assessed by bulk sampling, though 4.4 ± 1.9 × 10^3^ cells ml^−1^ as assessed from micromelt samples. While on the same order of magnitude, this is a roughly four-fold higher abundance from bulk as compared to micromelt techniques, with consistent differences apparent throughout (Fig. 3). We believe this discrepancy is driven by a methodological limitation of our micromelt sampling technique to liberate glacier algal cells from their icy environment. Using paired *in situ* microscopy, we have observed that glacier algal cells demonstrate a degree of adherence to their habitat and during this study were not readily captured by the force of the pump action. This adds weight to previous arguments that glacier algae likely employ mechanisms to maintain themselves within the constantly melting weathering crust (Irvine‐Fynn 2022). It seems likely that extracellular polymeric substance (EPS) production is the important, yet currently little studied (though see Doting et al. 2025) mechanism employed by glacier algae to this end (Fig. 3).

**Figure 4.**
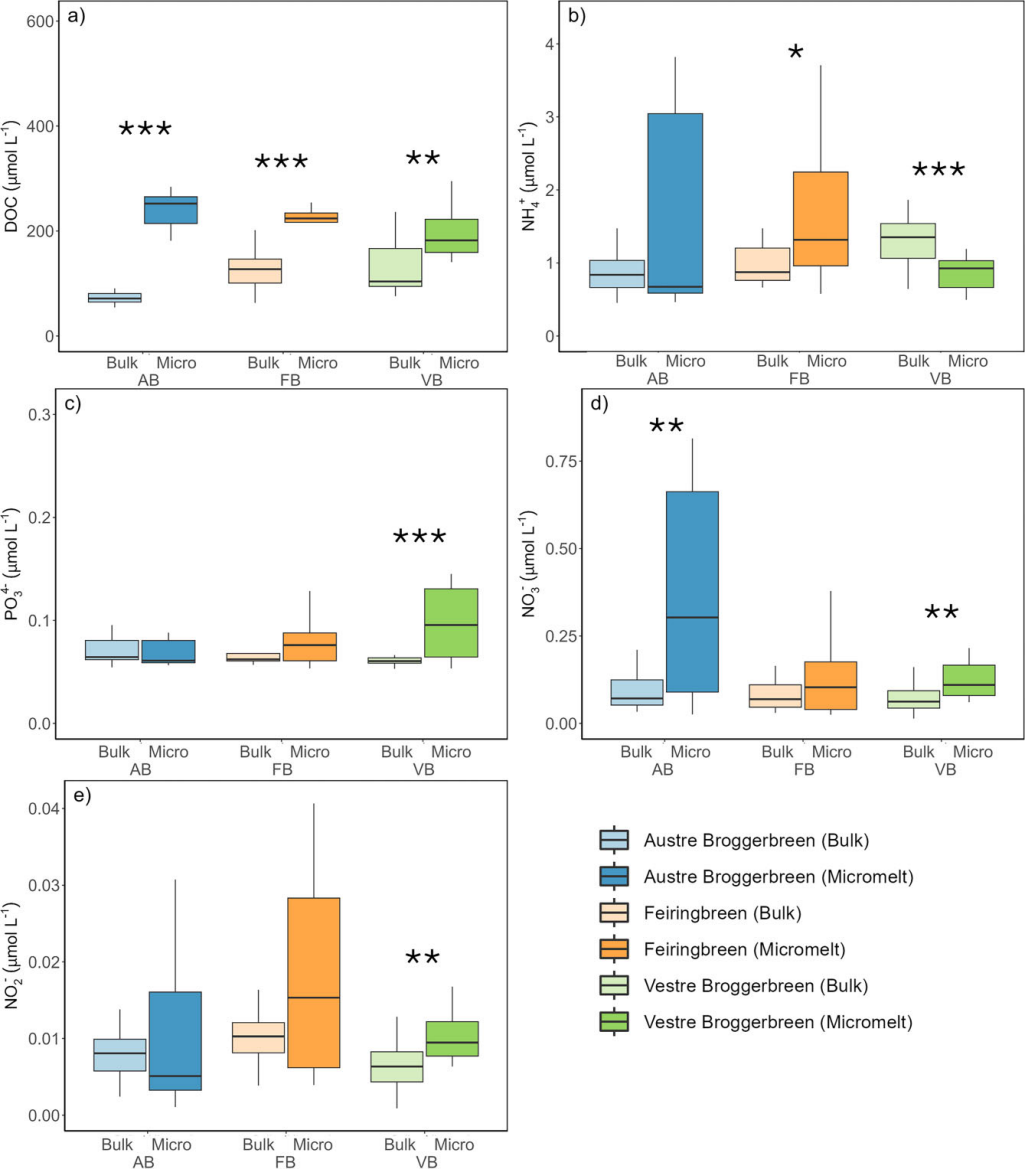
Comparison of major nutrient concentration within the aqueous geochemistry retrieved by traditional bulk and novel micromelt sampling methods. Supraglacial samples were taken from three Svalbard glaciers during the early ablation period (pooled timepoints, 06/2023): Austre Broggerbreen, Feiringbreen, and Vestre Broggerbreen. (a) DOC, (b) ammonium (NH_4_^+^), (c) phosphate (PO_4_^3−^), (d) nitrate (NO_3_^−^), (e) nitrite (NO_2_^−^). *n* = 10–15 excluding DOC (*n*_bulk_ = 6, *n*_micro_= 3). Significant difference between methods determined by Mann Whitney U, thresholds *P < 0.05, **P < 0.01, ***P < 0.001.

In contrast to glacier algal cellular abundance outcomes, sampling revealed differences in the inorganic nutrient, major ion and DOC concentrations between micromelt samples and those derived from traditional bulk methods (Fig. 4). For inorganic macronutrients, micromelt samples generally contained increased concentrations of ammonium (NH_4_^+^), nitrate (NO_3_^−^), nitrite (NO_2_^−^), and phosphate (PO_4_^3−^), though trends were not uniform, and higher micromelt concentrations observed here remained well within oligotrophic levels. Overall, bulk sampling methods returned lower and relatively consistent concentrations of inorganic macronutrients across our three glaciers and two sampling timepoints, with concentrations averaging 1.21 ± 0.7 µmol L^−1^ NH_4_^+^, 0.087 ± 0.06 µmol L^−1^ NO_3_^−^, 0.008 ± 0.003 µmol L^−1^ NO_2_^−^, and 0.074 ± 0.033 µmol L^−1^ PO_4_^3−^ across all bulk data. In contrast, micromelt inorganic macronutrient concentrations were more heterogenous across glaciers and time points, and for NH_4_^+^, NO_2_^−^, and PO_4_^3^, tended to show significant differences between sampling time points on the same glaciers (Fig. 4), with overall concentrations averaging 1.48 ± 0.99 µmol L^−1^ NH_4_^+^, 0.249 ± 0.248 µmol L^−1^ NO_3_^−^, 0.015 ± 0.012 µmol L^−1^ NO_2_^−^ and 0.092 ± 0.053 µmol L^−1^ PO_4_^3−^ across micromelt data. Whilst it is hard to draw definitive conclusions from these data on the inorganic macronutrient environment experienced by glacier algal cells within their meltwater film, our data indicate that this nutrient environment is likely to be more heterogenous and higher in concentration than expected from bulk sample analyses. This is consistent with previous assertions that glacier algae do not appear to be inorganic nutrient limited within ablating surface ice (e.g. McCutcheon et al. 2021, Millar et al. 2024, Halbach et al. 2025), though given that our micromelt concentrations remain well within the range considered oligotrophic (i.e. < 5 µmol L^−1^ N and < 0.5 µmol L^−1^ PO_4_), it seems likely that the ability of glacier algae to proliferate in surface ice may not relate to significantly enhanced nutrient availability in their thin meltwater film but rather rapid cellular uptake and/or lower cellular macronutrient requirements (Holland et al. 2022, Halbach et al. 2025). The heterogenous nature of the micromelt sampling observed here is also in line with stoichiometric analysis of single glacier algal cells, revealing a highly heterogeneous C:N and C:P ratios on a cellular level, potentially suggesting a flexibility in the acclimation of these cells to their micro-environment (Halbach et al. 2025). As sampling for this study proceeded during very early blooms stages across our Svalbard field sites, it would be interesting to examine whether micromelt macronutrient fractions remain consistent at later stages of high abundance blooms, which were not characterised here.

Several major ion species were significantly increased in micromelt fractions as compared to bulk samples during this study. Ice solute exclusion and preferential elution during freeze-thaw cycles provide a putative *in situ* concentrating mechanism, whilst aeolian deposition of mineral dust and marine aerosols may be important exogenous influences on the micromelt geochemical signature and thus glacier algal habitat (Kjær et al. 2015, Bergstrom et al. 2023). Sodium (Na^+^) and chloride (Cl^−^) ions together accounted for over 90% of the ions measured across all locations and time points, and were significantly increased in micromelt at approximately double their bulk concentrations (Table 1). The same pattern was also apparent for potassium (K^+^), showing lower but approximately double concentrations in micromelt as compared to bulk samples, while calcium (Ca^2+^), magnesium (Mg^2+^), and sulphate (SO_4_^2−^) were present at lower concentrations and remained consistent between sample methods (Table 1).

**Table 1.** Major ion concentration recovered from two supraglacial sampling methods tested upon three glaciers in Svalbard.

a) Bulk sampling		Na^+^	Cl^−^	Ca^2+^	Mg^2+^	K^+^	SO_4_^2−^
**Svalbard average (2024)**	**Range**	53.8–345	75.4–170	0–260	0–90	0–183	0–146
	**Mean ± SD**	256±31.7	115±13.7	27.7±56.4	10.5±15.7	3.09±0.93	4.5±20.4
	**EF**	1.29	6.32	0.57	1.49	0.38	6.32
	***n***	70	71	70	69	70	71
**Austre Broggerbreen**	**Range**	242–354	85–170	0–61.6	0–36.5	2.36–5.25	0–4.03
	**Mean ± SD**	270±23.2	121±17.5	9.21±17	6.81±10.6	3.1±0.664	0.859±0.925
	**EF**	1.3	2	0	1.42	0.07	2
	***N***	20	20	20	20	20	20
**Feiringbreen**	**Range**	26.9–146	75.4–125	3.66–175	0–90	0–5.33	0–146
	**Mean ± SD**	119±5.27	108±12.9	33.1±45.4	15.3±22.5	2.93±0.945	14.4±37.3
	**EF**	1.28	8.04	0.72	1.51	1.29	8.04
	***n***	20	20	20	20	20	20
**Vestre Broggerbreen**	**Range**	105–140	84.5–128	0–260	0–183	2.4–8.57	0–2.18
	**Mean ± SD**	129±1.38	115±9.29	36.4±75.4	15.5±33.8	3.19±1.08	0.476±0.384
	**EF**	1.3	8.33	0.68	1.54	0	8.33
	***n***	30	30	30	30	30	30
**b) Micromelt sampling**		**Na^+^**	**Cl^−^**	**Ca^2+^**	**Mg^2+^**	**K^+^**	**SO_4_^2−^**
**Svalbard average**	**Range**	64.4–2342	24–1039	0–72.1	0–23.1	1.4–14.8	0–7.02
	**Mean**	645±570	293±263	26.8±21.9	9.59±5.66	6.01±3.85	1.64±1.2
	**EF**	1.28	2.41	0.17	1.14	0.05	2.41
	***n***	51	51	51	51	51	51
**Austre Broggerbreen**	**Range**	71.6–2342	24–139	17.1–49.3	0–23.1	1.6–12.8	0–4.56
	**Mean**	710±755	318±338	33.6±9.98	14.5±5.47	5.69±3.78	1.92±0.929
	**EF**	1.3	2.78	0.23	0.99	0.06	2.78
	***n***	15	15	15	15	15	15
**Feiringbreen**	**Range**	64.4–1549	25.1–749	0–72.1	0–15.7	1.4–14.8	0–7.02
	**Mean**	620±462	279±214	37.1±23.4	10.6±2.29	6.4±4.08	1.56±1.51
	**EF**	1.29	3.51	0.19	1.27	0.05	3.51
	***N***	22	22	22	22	22	22
**Vestre Broggerbreen**	**Range**	246–1807	113–887	0–9.01	0–6.48	3.27–13.2	0–3.65
	**Mean**	613±534	289±262	3.19±3.23	0±0.244	5.72±3.78	1.46±0.912
	**EF**	1.23	0.29	0	1.1	0.05	0.29
	***n***	14	14	14	14	14	14
	**LoD**	**1.97**	**0.94**	**215.74**	**7.3**	**0.57**	**0.88**

(a) Bulk (liquid + ice matrix) and (b) micromelt (liquid fraction). Ranges represent the minimum and maximum concentration (µeqL^−1^) and mean ± standard error (µeqL^−1^) of both timepoints, EF shows the enrichment factor of each major ion relative to Cl^−^ in seawater (see methods aqueous geochemistry), and *n* denotes the sample number.

Given the proximity of our sampled glaciers to Kongsfjord (Fig. 1), it is likely that marine aerosols were responsible for the enhanced micromelt Na^+^ and Cl^−^ concentrations recorded here (Bergstrom et al. 2023). Previous studies have consistently demonstrated the influence of marine aerosols on glacial surface ice geochemistry, with Bagshaw et al. (2013) and Bergstrom et al. (2023) highlighting increasing Na^+^ and Cl^−^ concentrations across glaciers of Tyndall Valley, Antarctica, with proximity to the coast. Higher Na^+^ and Cl^−^ concentrations apparent during this study as compared to these previous works (approximately 6 to 20 times greater) likely reflected the immediate proximity of our sampling sites to open water, with all other solutes showing highly comparable ranges (e.g. compare Table 1 here with table 2 of Bagshaw et al. 2013). Our data thus demonstrated that glacier algae may experience higher salinities within their micromelt habitat on glaciers located more proximal to marine environments. Most photosynthetic organisms show a significant decrease in photosynthetic activity under high salt stress related to reactive oxygen species production and diversion of cellular resources away from PSII protein repair toward maintenance of wider cell homeostasis (Shetty et al. 2019). Jensen et al. (2024) recently demonstrated a decrease in glacier algal maximum quantum yield in the dark-adapted state (Fv/Fm) from 0.41 to 0.29 with a 5% salinity increase above ambient Greenland Ice Sheet conditions (i.e. 0%), though no further decline in Fv/Fm up to 15%. This, coupled with the widespread prevalence of glacier algal blooms across our sampling sites and many other marine adjacent glaciers, suggests that glacier algae hold potential to tolerate enhanced salinities within their micromelt environment (Remias 2012).Additional to marine aerosol contributions to ionic signatures, estimation of mean enrichment factors for major ion species relative to seawater (see methods) highlighted the importance of other sources of solutes to our samples. Both bulk and micromelt samples were enriched in Ca^2+^ > K^+^ relative to sea salt, consistent with previous observations from glacial systems (Fortner et al. 2005, Bagshaw et al. 2013). These ions are derived from the dissolution of dust during ice melt (Lyons et al. 2003), which was abundant in all samples of this study and is commonly reported as co-varying with glacier algal abundance (e.g. Williamson et al. 2019). Enrichment factors of Ca^2+^, particularly associated with the presence of terrestrial dust (Lyons et al. 2003), were notably higher in bulk (6.12 ± 3.57) as compared to micromelt samples (2.19 ± 1.68). Bulk sample ionic signatures were thus more enriched with terrestrial derived ions, in comparison to micromelt fractions that better reflected marine and other wind-borne inputs to the system. Given known spatiotemporal variation in marine aerosol and aeolian inputs to glacial surface ice (Bagshaw et al. 2013, Bergstrom et al. 2023), glacier algae likely experience a highly heterogenous microenvironment across their distribution relative to individual glacier ecologies including, proximity to marine environments and anthropogenic disturbances, surrounding land cover, and melt dynamics. Deciphering the associated impacts to glacier algal physiology and bloom dynamics requires dedicated sampling through space and time across myriad glacial settings paired with assessment of glacier algal responses.

Our data further demonstrated that glacier algae possess the ability to impact the geochemical signature of their micromelt habitat themselves through production of dissolved organic substances. Previous bulk-based studies have shown a progression from inorganic to organic dominated aqueous geochemistry associated with glacier algal blooms (Holland et al. 2019). In this study, micromelt samples demonstrated enhanced DOC concentrations from the onset of sampling, with micromelt DOC significantly higher (MWU *P <* 0.001) at all sampling times and locations (AB_MICRO_ = 249.16 ± 55.3 µmol L^−1^, FB_MICRO_= 240 ± 42.9 µmol L^−1^, VB_MICRO_ = 197 ± 53.1 µmol L^−1^), as compared to bulk samples (AB_BULK_ = 81.6 ± 29.8 µmol L^−1^, FB_BULK_ = 132 ± 58.2 µmol L^−1^, and VB_BULK_ = 154 ± 118 µmol L^−1^, Fig. 4). Metabolic byproducts, photosynthates, remnants of cell lysis, and EPS production are common algal-derived organics that increase DOC in the surrounding substrate (Meiners et al. 2004, Ugalde et al. 2014). Recently, Halbach et al. (2025) demonstrated that Greenland Ice Sheet glacier algal communities rapidly release a large fraction of their assimilated inorganic carbon as DOC, which may represent an important carbon source to glacier surface microbial communities. In this study, microscopy observations taken during micromelt sampling also suggested EPS production as a means for glacier algal cells to attach themselves to the ice matrix itself, presumably helping to prevent loss from the system as surface melt proceeds. DOC concentrations recorded here in the micromelt of early glacier algal bloom stages were already at the higher end of those reported from bulk sampling for later bloom stages in Greenland (Holland et al. 2019), highlighting the higher affinity of our sampling method to quantify bloom-associated DOC cycling and demonstrating the consistent availability of DOC within the micromelt layer on melting glaciers colonised by glacier algae.

## Conclusion

Direct sampling of the thin micromelt film inhabited by glacier algal cells was undertaken here to examine whether the persistence of abundant glacier algal blooms within highly oligotrophic surface ice is in part due to enhanced nutrient and solute availability within their micro-environment. While our micromelt sampling technique was not sufficient to adequately capture glacier algal cells themselves, presumably due to adhesion to the ice matrix through polymeric substance production, the aqueous geochemistry (macronutrients, major ions, and DOC) of the micromelt layer did diverge from the geochemistry measured using traditional bulk-sampling techniques. Micromelt inorganic macronutrient concentrations showed heterogenous and in some instances elevated levels as compared to bulk sample concentrations, though remained within established oligotrophic parameters. Patterning in major ion concentrations reflected the strong influence of marine aerosols on the micromelt fractions of our sampled glaciers, likely exposing glacier algal communities to elevated salinities. In contrast, bulk sample ionic signatures better reflected the dominance of particulate-derived solutes. Higher DOC concentrations in micromelt fractions even at the early bloom stages sampled here revealed the provision of DOC to the microbial food web by glacier algal communities from the onset of bloom formation. Taken together, datasets suggest that the glacier algal micro-environment remains oligotrophic but more heterogenous and impacted by aeolian inputs than apparent when using bulk sampling techniques. Little knowledge currently exists on how glacier algal cells respond to such fine-scale heterogeneity across their surface ice environment.

## Funding

The authors acknowledge funding provided by the Leverhulme Trust (iDAPT RPG-2020–199 to C.W.), the National Environmental Research Council (CASP-ICE, NE/Y002636/1 to C.W.), and doctoral training funding of E.B. and M.L. from the University of Bristol.

## Supplementary Material

fiaf098_Supplemental_File
