# Supplementary material for: Micromelt sampling of the glacier algal nutrient environment
Source: FEMS Microbiol Ecol. 2025 Oct 6;101(11):fiaf098. doi: 10.1093/femsec/fiaf098 (PMC12525140; doi:10.1093/femsec/fiaf098)
Supplement: fiaf098_Supplemental_File [file fiaf098_supplemental_file.docx]

|  |  |  |  | Bulk samples (n) | | Micromelt samples (n) | |
| --- | --- | --- | --- | --- | --- | --- | --- |
| Location | **Date** | **Lat (decimel)** | **Long (decimel)** | **cellular abundance** | **aqueous geochemistry** | **cellular abundance** | **aqueous geochemistry** |
| Vestre Brøggerbreen | 06-Jul | 78.914196 | 11.754798 | 18 | 22 | 5 | 5 |
| Vestre Brøggerbreen | 19-Jul | 78.912457 | 11.74668 | 10 | 10 | 9 | 9 |
| Feiringbreen | 10-Jul | 79.006498 | 12.447865 | 9 | 10 | 10 | 14 |
| Feiringbreen | 22-Jul | 79.006667 | 12.446944 | 10 | 10 | 8 | 10 |
| Austre Brøggerbreen | 14-Jul | 78.897387 | 11.833194 | 7 | 10 | 10 | 10 |
| Austre Brøggerbreen | 25-Jul | 78.896944 | 11.841667 | 9 | 10 | 5 | 5 |

Supplementary Table 1. Site locations on each glacier and sampling replication details for collection type.
